# Using a discrete choice experiments to explore societal preferences for valuing new drugs for rare diseases

**DOI:** 10.1186/s13023-025-04141-0

**Published:** 2025-12-29

**Authors:** Constanza Vargas, Stephen Goodall, Deborah J. Street, Manuel Espinoza, Richard De Abreu Lourenço

**Affiliations:** 1 Centre for Health Economics and Evaluation, University of Technology Sydney, Building 20, 100 Broadway, Chippendale, Sydney, NSW 2008 Australia; 2 School of Public Health, LKS Faculty of Medicine, University of Hong Kong, Hong Kong, Hong Kong

**Keywords:** Rare disease, DCE, Preferences

## Abstract

**Background:**

Rare diseases affect few people, but collectively they affect a substantial proportion of the population. Limited treatment options and the additional challenges of securing public funding make reimbursement decision making particularly complex, mainly due to the inherent evidence uncertainty, lack of understanding of these diseases and the greater impact on non-health outcomes. This study explored societal preferences regarding which factors should be valued when considering public subsidy of drugs to treat rare diseases.

**Methods:**

A discrete choice experiment was developed, and respondents were asked to assume the role of a Government advisor and decide on funding between 2 drugs to treat a rare disease. Attributes were identified from the literature and focus groups, including uncertainty of the evidence, unmet need, magnitude of the clinical benefit, magnitude in quality of life and total cost to government. A representative sample of Australians (*n* = 1099) completed the online survey. Data were analysed using mixed logit regression and latent class models to examine heterogeneity. Willingness to pay was also estimated.

**Results:**

In general, respondents had a greater preference for drugs that increase survival, where there was greater confidence in the effectiveness of the new drug and which increased patients’ capacity to do their usual activities. Preferences were not homogenous, the latent class analysis identified three groups: Class 3 (58%) demonstrated a strong preference for improvements in survival; Class 2 (21%) showed a strong preference for confidence in the evidence; and Class 1 (21%) positively valued increased government expenditure.

**Conclusion:**

These results are consistent with previous studies that used different methodologies in showing a preference for drugs with improved survival and quality of life. However, addressing a societal preference for greater confidence in the evidence - reducing evidential uncertainty - represents a methodological and policy challenge for the evaluation of drugs in rare diseases.

**Clinical trial number:**

Not applicable.

**Supplementary Information:**

The online version contains supplementary material available at 10.1186/s13023-025-04141-0.

## Background

Individually, rare diseases affect few people, but collectively they affect a substantial proportion of the population. The issue of low patient numbers impacts the availability of drugs for rare diseases in two ways: [1] the pharmaceutical industry has limited incentive to invest in research and development (R&D) of new treatments given the lower return on investment and; [2] regulatory challenges due to the difficulties in demonstrating effectiveness [1].

Once a drug achieves regulatory approval and enters the market, one challenge that arises is how to make the drug accessible to patients. Many countries have implemented health technology assessment (HTA) as a process that aims at determining the value of a new health technology (e.g. a new drug) to inform reimbursement decision-making with the ultimate aim of ensuring an equitable, efficient, and high-quality health system [2, 3]. This is particularly challenging in the context of reimbursement of drugs for rare diseases, where achieving efficiency, measured as value for money, is rarely possible, meaning treatments are less accessible raising significant equity concerns [4, 5]. Estimating value of any new drugs for a rare disease is more challenging because of the substantial/greater uncertainty of the evidence arising from the extremely low patient numbers enrolled in trials, lack of understanding of the underlying disease which directly impacts on the identification of relevant clinical outcomes, inadequate access to appropriate care, social isolation, and various other challenges [6]. The factors influencing decision-making must be clearly identified and carefully balanced. The introduction of any new technology will inevitably result in some patients being denied access to other technologies due to resource constraints. This challenge is even more pronounced in the case of rare diseases, where the higher cost per patient leads to a greater opportunity cost [7, 8].

Efforts have been made in the international community to address some of the evidence evaluation challenges of rare diseases in HTA. For example, in 2022 the European HTA Regulation (EU 2021/2282) introduced joint clinical assessments (JCAs) across EU Member States with the aim to harmonise the evaluation of the clinical value of oncology products and advanced therapy medicinal products on a first phase (starting in 2025) and orphan designation products as of 2028. This initiative represents a significant step towards integrating rare disease specific considerations into HTA, fostering greater consistency in evidence requirements. Nonetheless, much remains to be done, as approaches to assessing access to treatments for rare diseases continue to vary widely across jurisdictions, with some processes operating outside the established principles of HTA. This was confirmed by a systematic literature review that showed that most countries have adopted special considerations in their assessment processes to facilitate public reimbursement for rare disease drugs with many countries having more than one pathway available to access drugs for rare diseases [3].

Regardless of whether the decision-making process considers HTA, the pathway that leads to access will inevitably consider multiple factors to determine the value of the new drug. A systematic literature review identified 19 factors, or attributes, used as criteria in making decisions about reimbursement of new drugs for rare diseases [3]. Amongst the factors cited were health related quality of life, extent of therapeutic benefit and availability of other therapeutic options. In addition, severity, age at diagnosis, waiting times and side effects were also important [6, 7]. As a process that supports decision making, HTA considers many of these factors and determines the additional value of a new technology to inform reimbursement decisions. In the context of decision-making for rare diseases, gaining a deeper understanding of the factors that determine the value of a new drug requires exploring individuals’ preferences regarding what constitutes value in these new treatments.

Discrete choice experiments (DCEs) are widely used in healthcare research as they provide a robust methodology for eliciting preferences in situations where choices are required, such as in decision-making processes. Moreover, in the healthcare market - where revealed preference data is often unavailable, and prices do not necessarily reflect the true value for a new technology - stated preference methods like DCEs serve as a valuable tool for capturing these insights.

This study recognises that specific assessment pathways for rare diseases already exist for various reasons; among them, legitimate demands from society [9]. Therefore, the key question is not whether rarity itself should be prioritised, but rather which factors should determine the value of new drugs within these pathways. With this context, this study uses a DCE to explore the societal preferences for which factors should be considered in determining the value of new drugs for rare diseases in Australia and to estimate the marginal willingness to pay (WTP) for different attributes of these technologies.

## Methods

This study used a DCE to elicit societal preferences. DCEs are a stated-preference methodology particularly suited to this research, as it enables the exploration of societal preferences in a context where revealed data are unavailable. Furthermore, this method aligns with Lancaster’s economic theory, which posits that utility is derived from the characteristics of a good or service rather than the good or service itself [10]. Accordingly, the utility is based on the combination of attribute levels that describe a given option, which, in this study, refers to drugs for rare diseases. In practice, respondents are presented with hypothetical yet realistic scenarios in a survey and are asked to select their preferred alternative from a finite set of alternatives (also known as options). Each time that respondents are asked to make a choice from these options we say that they have completed a choice task. Each alternative is defined by different levels of pre-determined attributes that characterize a drug for a rare disease. By systematically varying the levels at which these attributes are presented, and analysing respondents’ choices, we can identify the attribute levels that are most important to them.

### Identification of relevant attributes

In this study the attributes and levels were obtained using three main sources:

1. a systematic literature review of assessment pathways of new drugs for rare diseases [3]. The review identified unique characteristics that were classified as targeting the process of decision-making, described a new methodology or were classified as attributes of value. Attributes from this literature review were deemed relevant if they related to the value of new drugs for rare diseases.
2. Targeted literature search in OVID (Medline and Embase) to specifically identify preference-based studies in the context of rare diseases. The targeted search used the terms “Discrete choice experiment* OR DCE AND rare disease*” and articles were included for data extraction if they reported results on a DCE or other stated preference methodology or articles that aimed at identifying attributes to inform a future preference-based study. Data were extracted on the preference-based method used, source of attributes, attributes (including the description), attribute levels, experiment design, mode of survey delivery if applicable, statistical analysis, use of opt out question and choice question.
3. Focus groups: two focus groups with three participants each were conducted with a multidisciplinary group of stakeholders that included clinicians/researchers, representatives of patient’s organisations and government representative. Participants were contacted via email which included an invitation containing a ‘Participant Information Sheet’ and ‘Consent Form’ (see supplementary material). All participants had at least some familiarity with the process of HTA and rare diseases in Australia. A convenience sampling technique was used to recruit participants with the aim of achieving saturation, particularly in identifying the relevant factors that determine the value of a new drug for rare diseases. The discussion was led by one experienced researcher (RDAL) following a script developed for the purpose of this focus group (Supplementary material). The second researcher (CV) ensured that pre-identified topics were raised or prompted during the discussion following a checklist. Recordings were transcribed, and a thematic analysis was conducted to identify relevant themes and the development of candidate attributes. Participation in the focus groups was voluntary, and no payments were made to participants.

### Final list of attributes

A tentative list of 35 attributes and levels were identified, synthesized and discussed within the research team A list of 6 candidate attributes with corresponding descriptions and levels was presented to a group of DCE experts within the Centre for Health Economics Research and Evaluation (CHERE) at the University of Technology Sydney (UTS). During this session the comprehensibility of the attributes was discussed and as a result some attributes were combined, and the wording of the attributes/levels description was improved.

The final list of attributes and levels included in the survey is shown in Table 1.

**Table 1:** Attributes and levels included in the DCE survey

Attributes	Description	Levels
Uncertainty of the clinical evidence	The degree of confidence in the clinical evidence is described as:	• Not confident that it works as there is very limited data on efficacy and safety. • Confident that it works as there is strong evidence on efficacy and safety.
High unmet need	The extent to which alternative treatments for the rare disease are available.	• Effective alternative treatments are available • Other treatments are available but are of limited effectiveness. • No other treatment options are available.
Total cost of funding to Government for all patients per year	The total annual treatment cost to Government for all patients with the rare condition in Australia is:	• $1 million • $50 million • $100 million • $200 million
Magnitude of the clinical benefit	Extent to which the drug increases survival for the average patient	• No increase in survival • Increases survival by 1 year • Increases survival by 5 years
Magnitude of the benefit in quality of life with new treatment	The use of the new drug means that the patient:	• Will continue to be unable to do their usual activities, relying on a full time carer. • Will have moderate problems doing their usual activities, requiring some reliance on a carer. • Will have slight problems doing their usual activities, and will not require a carer. • Will have no problems doing their usual activities, and will not require a carer.

### DCE design

A full factorial design that includes all possible level combination of the attributes would translate into 288 items (1 attribute with 2 levels, 2 attributes with 3 levels and 2 attributes with 4 levels = 2^1^*3^2^*4^2^). Using choice tasks with two labelled options, the full number of choice tasks possible would be equal to 41,328 (288*287/2). Therefore, a fractional factorial design was implemented to efficiently measure only the main effects while ensuring that the effects of interest could be accurately estimated [6]. The design included 128 choice sets, which were then structured using a generator-developed design into 16 blocks (versions), each containing 8 choice sets [11]. Respondents were randomly assigned to complete one of these blocks and the sequence of choice sets within each block was also randomised. The design did not allow for an opt-out-option. In the context of this research, this was considered unnecessary, as in real world reimbursement decisions, choices are always made regardless of their difficulty or whether neither option is deemed appealing. Some restrictions (see Supplementary material) were imposed in the presentations of attributes and levels and some overlap of attribute levels was allowed to stop respondents from choosing based only on one attribute throughout the whole survey.

### Data collection

Data was collected via an online survey hosted by PureProfile sent to adult participants representative of the Australian population in terms of age, gender and region. Participants were recruited by Pureprofile (https://www.pureprofile.com/) with quota limited by age, gender, and state ensuring the sample was to be representative of the Australian general population. Data was cross checked by PureProfile and excluded responses that were completed in less than 30% of the median response time or by responders with nonsensical answers (i.e., not words or answer does not answer question) to open-ended questions. Additionally, a CAPTCHA was used to remove bots. An initial pilot sample was completed with 113 respondents to check comprehensibility of the survey and the ability to fit the desired models. Some post-choice questions that assessed the clarity of each attribute’s definition were removed, as the pilot results indicated that respondents found the attributes to be clear. The final sample target was 1,000 complete responders meaning that the survey provided at least 62 observations per choice set. Analysed data was quality checked by PureProfile and excluded: [1] responders completing the survey in less than 30% of the median time of response; and [2] responders who had nonsensical responses in the open ended questions. In addition, captcha was used to remove bots.

### Survey development

The survey consisted of a background section which introduced the concept of rare diseases, which is the focus of this study (see Box 1), the challenges associated with (funded) access to new drugs for rare diseases and the process for drug reimbursement in Australia (the survey can be accessed in the Supplementary material). This section was followed by a brief description of the attributes and the choice vignette which described the role of the respondent and baseline setting for which choices were to be made.

Box 1. Rare disease definition for the context of this DCE.

**Table:** 

A rare disease is one that affects more than 1 patient and less than 5 in 10,000 people. Most rare diseases, 80%, have a genetic origin, 50% affect children and in 30% of cases the patient will die before the age of 5. Despite being very different in their presentation, they are generally known for being severe and having a negative impact on the patient, family and carer’s quality of life.
The natural course of life gets altered as well as the day-to-day life activities such as work, social engagement and participation, hobbies, and even basic self-care may become challenging, often necessitating the involvement of caregivers.

**Fig. 1:**
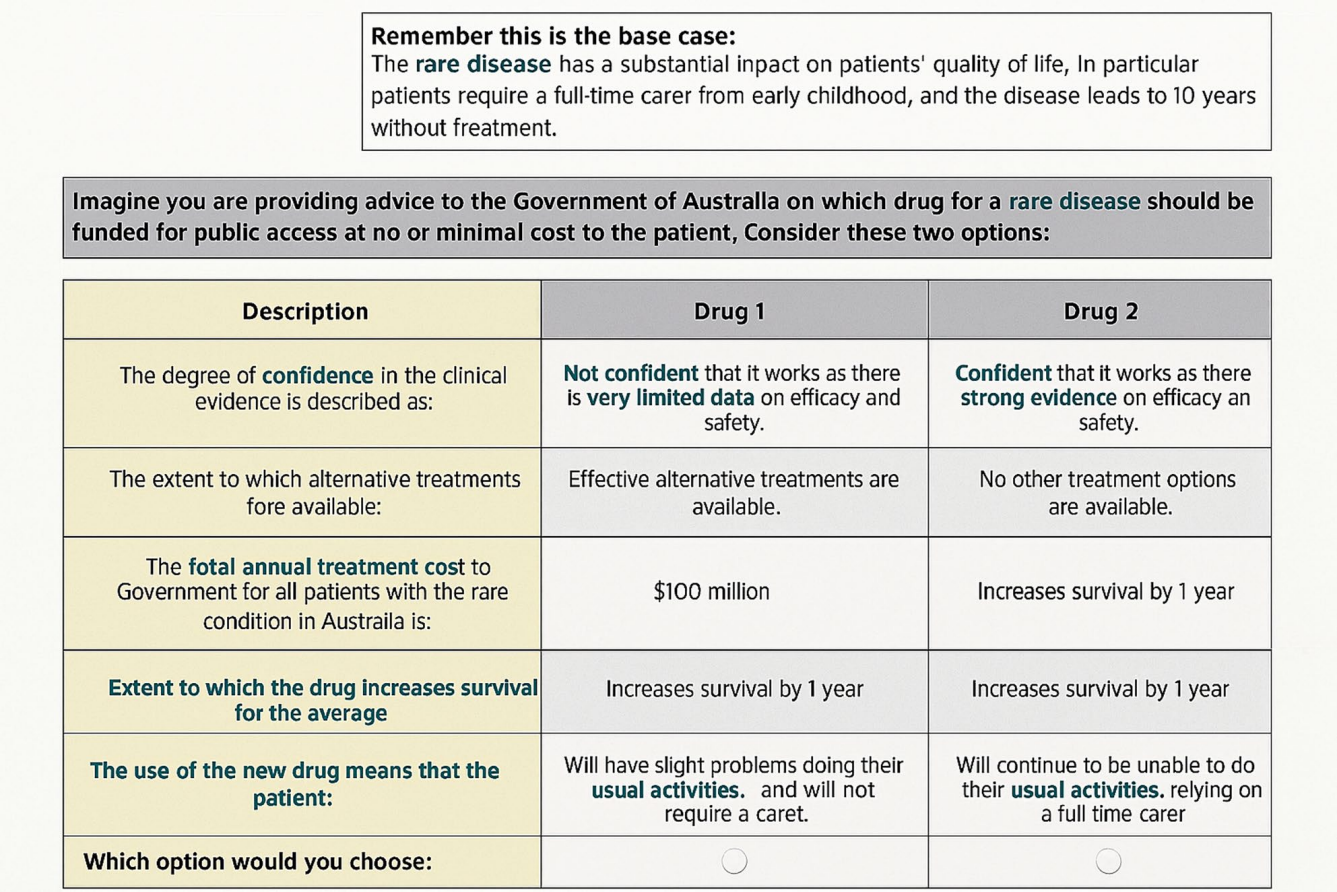
Choice task example

At the end of the survey a question was asked regarding the attribute that was most and least important. In addition, general questions regarding the comprehensiveness of the survey were asked after the choice set was completed followed by questions on the respondent’s demographics. The survey was loaded, hosted, and managed using the PureProfile online platform. An example of a choice task is presented in Fig. 1.

### Statistical analysis

The choice data were analysed using a conditional logit model (which accounts for the individual heterogeneity in the responses), a mixed logit model that allows for the random variation in preferences across the respondents and a latent class model to explore those characteristics of the individuals that may explain these differences in preferences. The marginal willingness to pay (WTP) was estimated using the results from the mixed logit analysis assuming a normal distribution for the coefficients; the WTP for each attribute was calculated as the ratio of the attribute’s coefficient to that of the total cost to Government attribute (expressed as a continuous variable) [12]. The Wald test indicated non-linearity of the survival attribute (*p* < 0.000), supporting the use of categorical coding of this variable in the model.

Determination of the optimal number of classes (maximum of 10) for the latent class was based on the lowest Consistent Akaike’s Information Criterion (CAIC) [13]. Respondents’ demographics used as covariable to run the latent class model were sex, age, income, education level, health literacy, experience as a health care decision maker, experience/knowledge on rare diseases, being a parent and experience as a carer.

All statistical analysis were conducted in STATA 18 using the clogit, mixlogit and lclogit2 commands for the conditional logit, mixed logit and latent class models, respectively [14, 15].

This study obtained ethical approval from UTS human research ethics committee (HREC) (UTS HREC ETH21-6090). The results of this DCE are reported following the DIRECT checklist, which is presented in the Supplementary material [16].

## Results

A total of 1,099 respondents completed the survey. The characteristics of the respondents are presented in the Supplementary material (Table S1). Overall, the sample is reflective of the Australian general population with respect to age, gender, health status and geographic distribution. However, the sample shows a higher proportion of respondents with higher educational levels.

**Fig. 2:**
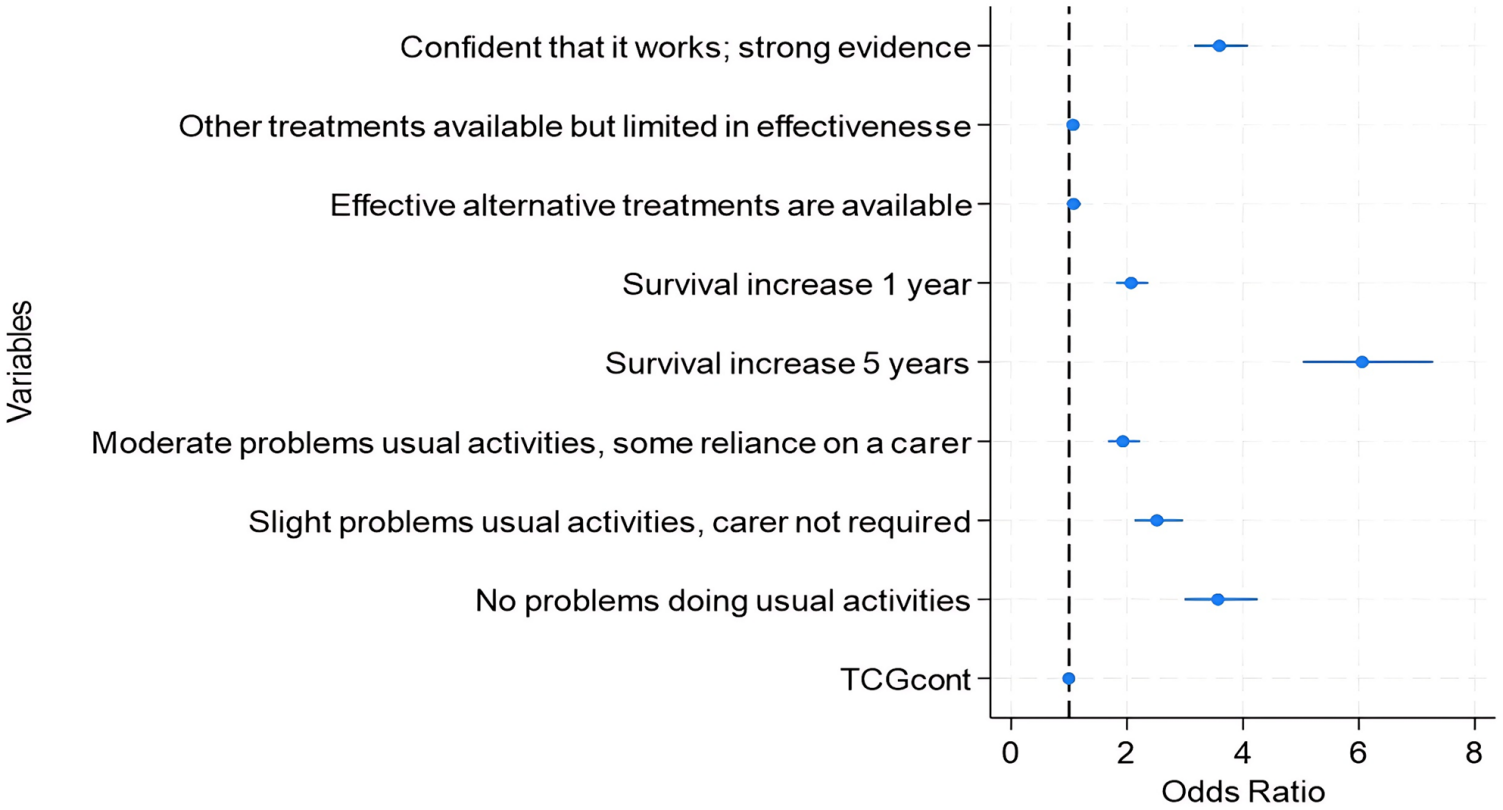
*Mixed logit model results.* Abbreviation: TCG, total cost to Government. Note: The figure presents exponentiated coefficients, expressed as odds ratios. Accordingly, negative coefficients correspond to odds ratios below 1

The results of the conditional logit and mixed logit models are presented in Table 2, with a visual representation of the results of the mixed logit in Fig. 2 (see Figure S1 in the Supplementary material for the conditional logit model).

**Table 2:** Conditional logit and mixed logit analysis

Attributes	Levels	Conditional logit	Mixed logit with cost variable continuous	
		Coefficient (SE)	Coefficient (SE)	SD (SE)
Uncertainty *Not confident that the new drug works is omitted*	Confident that it works as there is strong evidence on efficacy and safety	0.874 (0.036)***	1.279 (0.066)***	1.116 (0.073)***
Unmet need *Effective Tx are available*	No other Tx are available	-0.036 (0.042)	0.064 (0.046)	-0.003 (0.016)
	Other Tx are available but their effectiveness is limited.	0.045 (0.034)	0.072 (0.058)	-0.436 (0.137)**
Magnitude of clinical benefit *No increase in survival is omitted*	Survival increases by 1 year	0.495 (0.045)***	0.727 (0.068)***	-0.055 (0.065)
	Survival increases by 5 years.	1.210 (0.053)***	1.801 (0.094)***	1.137 (0.078)***
Magnitude in quality of life *Unable to do usual activities is omitted*	Moderate problems doing their usual activities, requiring some reliance on a carer.	0.427 (0.052)***	0.656 (0.073)***	-0.058 (0.061)
	Slight problems doing their usual activities (no carer needed)	0.644 (0.061)***	0.922 (0.084)***	0.15 (0.287)
	No problems doing their usual activities (no carer needed)	0.849 (0.060)***	1.272 (0.089)***	-0.391 (0.194)
Total annual cost to Government *$1 M is omitted*	$50 M	-0.323 (0.044)***	-0.007 (0.000)***	-0.009 (0.001)***
	$100 M	-0.631 (0.053)***		
	$200 M	-0.919 (0.060)***		
**Statistics**				
Log pseudolikelihood		-4878.897	-4646.855	
AIC		9779.794	9329.711	
BIC		9865.316	9469.656	

Abbreviations: AIC, Akaike information criterion; BIC, Bayesian information criterion; M, million; NA, not applicable; SE standard error; SD, standard deviation; Tx, treatment

Notes:

Statistical significance is denoted by (***) at the 1% level, by (**) the 5% level and by (*) the 10% level

*Text in italics corresponds to the base level*

### Conditional logit analysis

The conditional logic model shows that all coefficients are positive, except for the no treatment available (though not statistically significant) and cost to government which are negative. A positive attribute coefficient indicates that, as the coefficient of that attribute level increases, respondents are more likely to choose one scenario over another. The larger the coefficient, the greater the attribute level’s influence on their decision. In particular, the coefficients for each level of the total cost to Government attribute show that the difference between them are very similar showing linearity which was confirmed via the Wald test.

The attribute that showed the strongest predictor of preference, as assessed by the magnitude of the coefficient, was the capacity of the new treatment to improve survival closely followed by the confidence in the clinical evidence that the new drug works and the impact the new drug has on the ability of patients to do their usual activities.

Respondents appeared indifferent to unmet need, which, in the context of this research, referred to whether other treatments of similar or lesser effectiveness were available (*p* = 0.805 and 0.381 for treatments with limited effectiveness and no treatments available, respectively, in the conditional logit model).

### Mixlogit regression model

Results of the mixed logit show that respondents had a greater preference for the impact of the new drug on survival closely followed by the confidence in the clinical evidence that the drug works and the impact of the new drug in the patients’ capacity to do their usual activities (Table 2). Of these, the largest coefficient was observed in the highest survival (5 years) versus no improvement in survival. Of the nine standard deviations estimated, four were statistically significant at the 5% level, indicating that there is heterogeneity in at least one level in 4 of the 5 attributes. This finding suggests heterogeneity among respondents concerning their preferences for the uncertainty regarding the clinical evidence, the clinical benefit in terms of survival, the unmet need and the total cost to the government.

Table 2 here (included after the references).

### Latent class analysis

The optimal number of classes for the latent class analysis was 3 (detailed results in Table S2 of the supplementary material). Results for the latent class analysis are provided in (Table 3). Class 1 (21%) included respondents who did not have strong preferences for any the attributes presented, however a few aspects stand out. Firstly, compared to Classes 2 and 3 they preferred new drugs for rare diseases with higher total cost to Government. This class also had a stronger preference for drugs that improve patient’s ability to do their usual activities more than the increase in survival. In contrast, Class 2 (also 21%) included respondents with the strongest preference for the certainty around the clinical evidence followed by drugs that improve survival. To a lesser extent, this Class also had a preference for drugs that had other treatments available, but their effectiveness was limited. Finally, the majority of respondents were in Class 3 (58%) who had strong preferences against new costlier drugs followed by new drugs that improve survival and new drugs that improve a patient’s ability to do their usual activities.

**Table 3:** Latent class analysis (three classes)

Logistic regression results for class assignment		Class 1	Class 2	Class 3
	Constant	0.239	-2.23	
	Gender	0.2	0.118	
	Health status	0.356	-0.118	
	Health literacy	-1.042	0.813	
	Decision maker	-0.002	0.067	
	Income	-0.349	0.637	
	Education	-0.574	0.016	
	Familiarity with rare diseases	0.078	0.026	
	Being a parent	-0.448	-0.095	
	Being a carer	0.918	-0.591	
	Class share	21%	21%	58%
**Attributes estimates by class**		**Class 1**	**Class 2**	**Class 3**
Uncertainty Not confident that the new drug works is omitted.	Confident that it works as there is strong evidence on efficacy and safety	0.2291*** **(0.076)**	3.4139*** **(0.3428)**	0.8858*** **(0.0695)**
Unmet need Effective Tx are available	Other Tx are available but their effectiveness is limited.	0.0723 (0.0912)	0.5694** **(0.2268)**	-0.1236* **(0.0657)**
	No other Tx are available	-0.1101 (0.1002)	0.1798 (0.2543)	-0.0152 (0.0739)
Magnitude of clinical benefit No increase in survival is omitted	Survival increases by 1 year	0.1501 (0.1202)	0.7303** (0.2885)	0.9194*** **(0.0866)**
	Survival increases by 5 years	0.2756** (0.1303)	1.2912*** (0.2565)	2.2279*** **(0.1258)**
Magnitude in QoL Unable to do usual activities is omitted	Moderate problems doing their usual activities, requiring some reliance on a carer.	0.1544 (0.1265)	0.7213 (0.2602)	0.7959*** **(0.0891)**
	Slight problems doing their usual activities (no carer needed)	0.2982 (0.1511)	1.1933 (0.314)	1.1652*** **(0.1027)**
	No problems doing their usual activities (no carer needed)	0.3691** **(0.1495)**	1.7842 (0.3218)	1.4198*** **(0.1067)**
Total annual cost to Government $1 M is omitted	$50 M	0.1867 (0.117)	-0.5046 (0.2481)	-0.7722*** **(0.0903)**
	$100 M	0.41*** **(0.1393)**	-0.7971 (0.3696)	-1.458*** **(0.1112)**
	$200 M	0.3266** **(0.1459)**	-0.9122 (0.3514)	-2.0131*** **(0.1347)**
	Gender	0.2023 (0.2186)	0.1216 (0.2046)	NA
	Health status	0.3548 (0.3186)	-0.1222 (0.2733)	NA
	Health literacy	-1.0454** **(0.4695)**	0.8103 (0.844)	NA
	Decision maker	-0.001 (0.2473)	0.0703 (0.2298)	NA
	Income	-0.3468 (0.2289)	0.6448** **(0.2501)**	NA
	Education	-0.5762** **(0.2354)**	0.0117 (0.2405)	NA
	Familiarity with rare diseases	0.0769 (0.3207)	0.0231 (0.3028)	NA
	Being a parent	-0.4481** **(0.2188)**	-0.0926 (0.2037)	NA
	Being a carer	0.9193** **(0.3441)**	-0.5942 (0.57)	NA
	Constant	0.2296 (0.6795)	-2.2463** **(0.9689)**	NA

M, million; NA, not applicable; QoL, quality of life; Tx, treatment

Standard errors in parentheses; **bold** text indicates statistically significant difference; *, significant at 10%; ** significant at 5%; *** significant at 1%

Relative to Class 3, Class 1 had statistically significantly more respondents who had carer responsibilities, fewer respondents who had children and had a lower educational and health literacy level (see supplementary material). In addition, relative to Class 3, Class 2 had more respondents with a higher level of income.

### Willingness to pay analysis

**Fig. 3:**
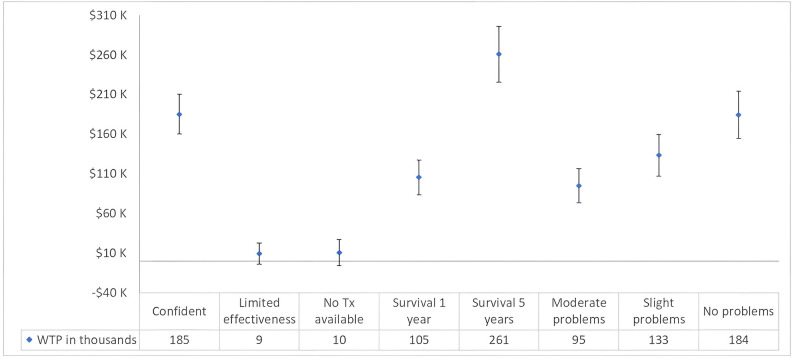
*Willingness to pay from the mixed logit analysis.* Abbreviations: K, thousands; Tx, treatment; WTP, willingness to pay

The mixed logit results show significance of the total cost to government attribute which enables the estimation of the marginal WTP. Results of the WTP from the mixed logit analysis are presented in Fig. 3. Respondents were willing to have the government spend an extra $259K for a new drug targeting rare diseases that increases survival by five years compared to a drug that has no increase in survival. Considering no increase in survival as the baseline, the willingness to pay is reduced to approximately an additional $104K if the new drug increases survival by 1 year. The WTP results also show that respondents are willing for the government to spend an additional $185K for new drugs for which there is confidence (reduced uncertainty) that the new drug works.

## Discussion

This study explored the general population’s preferences for which factors should be considered in the context of HTA decision making in Australia when assessing the value of a new drug for rare diseases. On average, respondents had a greater preference for drugs that increase survival, closely followed by greater confidence that the new drug works and increased benefit in the patients’ capacity to do their usual activities.

The existing literature on preferences relating to rare diseases mostly focuses on determining whether citizens prioritize the rarity of a disease, when forming resource allocation preferences. The attributes and levels, their sources, the way the choice question was framed, type of respondent (e.g. patient or general public) and the stated preference method used differed across the existing studies. In general, there is consistency across studies that rarity should not be prioritized over other relevant attributes such as severity [17–26]. The latter finding had a direct policy implication that translated in the acceptance of a higher incremental cost-effectiveness ratio (ICER) threshold in cases where more severe diseases are being assessed [19, 27]. This has shaped HTA structures in countries like England or Sweden where severity weighing is applied [28]. In England, severity is used as an eligibility criteria within highly specialised technology (HST) program for ultra-rare diseases. However, findings also show that in studies were rarity was not prioritized, a purely prevalence-based definition of rare diseases was used, which may fail to characterize appropriately what is a rare disease [17, 22, 26, 18]. Consequently, the way questions are framed in research studies may significantly influence the observed differences in findings or fail to accurately capture societal preferences in rare diseases [29].

In contrast, an Australian study revealed that many respondents support providing access to more expensive services for individuals with rare diseases, even if this came at the expense of reducing access to services that benefit a larger portion of the population [24]. The argument raised in this paper is that “fairness” in the context of reimbursement is determined by the comparison between the benefit received by a patient and the average cost borne by those who contribute to funding the treatment. In rare diseases, the total cost to government is relatively low, resulting in a low average cost for those sharing the financial burden. If this average cost is perceived to be sufficiently small in relation to the benefit received by the patient, the access of the new drug may be regarded as equitable [24].

Overall, our findings show that improvement in survival and confidence in the evidence supporting the drug’s effectiveness were key attributes for the average respondent [17, 30]. This is consistent with the available literature acknowledging the substantial differences across studies that make the results hard to compare (i.e., differences in the stated preference method design, survey structure, questions framing, rare disease definition and context around the challenges of rare diseases provided). Regardless, these two aspects are often lacking in the context of drugs for rare diseases. First, because enrolment of patients in clinical trials is extremely hard and consequently improvement in survival is generally not the outcome measure being pursued. The strong preference for improved survival suggests that the public prioritizes tangible health outcomes that significantly extend a patient’s lifespan, even more so than improvements in functional independence, such as the ability to perform usual daily activities without carer support. Second, evidence is frequently derived from very small trials and relying on surrogate endpoints, therefore largely uncertain. The preference for strong evidence may reflect a societal desire for transparency and accountability in the allocation of resources for rare diseases.

The results of the mixed logit and latent class analysis show that the preferences across respondents were not homogenous meaning that there are groups of individuals whose preferences may deviate from the average societal preference. This finding highlights the complexity of capturing diverse perspectives within a population. From a policy perspective, utility maximization is typically achieved by representing the preferences of the majority; however, it is equally important to acknowledge that some individuals’ preferences may not align with those of the broader population. Notably, education level, which were accounted for in the latent class analysis, was a distinguishing factor among the identified classes. In particular, relative to Class 3, individuals with more education were less likely to be in Class 1.

The latent class analysis identified three distinct classes. Class 3 demonstrated a strong preference for improvements in survival. Class 2 showed a strong preference for confidence in the evidence supporting the drug’s effectiveness. In contrast, Class 1 positively valued increased government expenditure which could reflect those respondents interpreted that increased total cost meant more access to treatments (i.e., that more people would be able to access treatment). While this interpretation could introduce bias, framing the attribute as total cost to the Government was intended to reflect the complex decision context faced by reimbursement authorities. An alternative framing, such as presenting costs as increases in household taxes, might have enabled a more direct estimation of the WTP. However, such an approach requires additional assumptions (e.g., regarding tax structures and distributional effects) that were not consistent with the aim of this study, which was to ask respondents to assume the role of a Government advisor.

The only DCE identified in the literature that assessed the heterogeneity of the responses via a latent class analysis was Mentzakies et al. [18]. This study identified 2 distinct classes based on the influence of cost on their choices: one where cost had a significant influence and another where cost had no influence. It is worth mentioning that the respondents in this study were a convenience adult population within a University setting, which may substantially differ to the adult general population. Moreover, two of the attributes considered in this DCE were related to cost (cost per patient and total cost to government) which required adjustment for interaction.

A finding from the conditional logit and mixed logit models was that respondents were largely indifferent to whether alternative treatments were available, despite “unmet need” being a common justification for prioritising rare diseases. This could be due to several reasons: firstly, that unmet need is an important attribute in prioritising rare versus non-rare conditions, which was outside the scope of this survey; secondly, that the attribute may have been framed in a way that implied some form of care would always be provided; thirdly, that the concept of “alternative treatments” may have been too abstract for the general public to interpret consistently; and finally, that respondents simply place greater weight on other attributes (e.g., quality of evidence or stakeholder involvement). However, the latent class analysis revealed that two of the three classes did show a significant (*p* < 0.05) and positive preference when alternative treatments were available but had limited efficacy. This suggests that unmet need may still play a role in shaping the preferences of what determines value of a new technology.

The results of our study also show that higher costs were less preferred compared to lower costs. The inclusion of “cost” as an attribute in these types of experiments may be considered controversial by some researchers [31]. This debate originates from the broader, unresolved discussion about what constitutes an attribute of value in the context of HTA, as there is no universal consensus on the definition of value in this field. Critics argue that cost represents a “loss” rather than a “gain,” and therefore does not inherently contribute to the value of a new drug. Furthermore, capturing the value for money (i.e. cost effectiveness) may lead to double counting as the value may already be accounted for in the benefit attributes.

However, in preference-based studies, we contend that cost is a critical attribute, as it is a factor considered by HTA decision-makers. In Australia, the structure of the HTA system places significant emphasis on the opportunity cost and the broader impact of new technologies on the national pharmaceutical budget. Although the Pharmaceutical Benefits Advisory Committee (PBAC) does not set the price of new drugs, it rigorously evaluates the cost per patient and the overall financial implications for the Australian government during the recommendation process. These considerations, along with other attributes, are assessed concurrently at each stage of the assessment pathway. Finally, we consider it critical to better understand what tax payers believe the government should or should not fund. This study provides additional insights into which criteria make sense in the space of rare diseases.

This study has several limitations that should be acknowledged. First, it is important to consider the inherent challenges associated with completing a task of this nature, which requires respondents to make choices between two options with which they are unlikely to be familiar. Such tasks demand an understanding of the complexities surrounding the funding of drugs, including opportunity costs, as well as the unique challenges associated with rare diseases. While the survey design was structured to provide as much contextual information as possible, efforts were also made to keep it concise to encourage respondents to carefully read and engage with the content. In this regard, some relevant aspects may have been missed. Furthermore, although a comprehensive approach was used to identify all relevant attributes, the final list had to be refined to a number that ensures the experiment was manageable for respondents. Inevitably, this could mean that we did not account for attributes that matter more to respondents than those that we included. Recruitment via a professional online panel, where participants are matched to surveys based on profiling questions and receive a small monetary incentive for completion, is a potential limitation. However, these methods are standard practice in health economics and outcomes research. To mitigate potential biases, our study sample imposed quotas for age, sex, and geographic distribution and was broadly representative of the Australian adult population. Recruitment via a professional online panel, where participants are matched to surveys based on profiling questions and receive a small monetary incentive for completion, is a potential limitation of the present study, although these methods are commonly used in health economics and outcomes research.

Overall, the results of this study were consistent with what the literature reports despite the different framing of the attributes and context provided to respondents. When determining the value of a new drug, respondents show strong preference for survival, certainty of the evidence and improvement in quality of life, all attributes that are hard to demonstrate when considering the reimbursement of treatments for rare diseases.

## Supplementary Information

Below is the link to the electronic supplementary material.

Supplementary Material 1

## Data Availability

The full survey is available in the Electronic Supplementary Material. The datasets analysed during the current study are not publicly but may be available from the corresponding author on reasonable request.

## Abbreviations

AIC: Akaike information criterion
BIC: Bayesian information criterion
CAIC: Consistent Akaike’s Information Criterion
CHERE: Centre for Health Economics Research and Evaluation
DCE: Discrete choice experiment
HREC: Human research ethics committee
HST: Highly specialised technology
HTA: Health technology assessment
ICER: Incremental cost-effectiveness ratio
K: Thousand
M: Million
NA: Not applicable
PBAC: Pharmaceutical Benefits Advisory Committee
R&D: Research and development
SD: Standard deviation
SE: Standard error
TCG: Total cost to Government
Tx: Treatment
UTS: University of Technology Sydney
WTP: Willingness to pay

## References

[1] Mulberg AE, Bucci-Rechtweg C, Giuliano J, Jacoby D, Johnson FK, Liu Q, et al. Regulatory strategies for rare diseases under current global regulatory statutes: a discussion with stakeholders. Orphanet J Rare Dis. 2019;14(1):36. 30736861 10.1186/s13023-019-1017-5 PMC6368795

[2] O’Rourke B, Oortwijn W, Schuller T. The new definition of health technology assessment: A milestone in international collaboration. Int J Technol Assess Health Care. 2020;36(3):187–90. 32398176 10.1017/S0266462320000215

[3] Vargas C, De Abreu Lourenco R, Espinoza M, Goodall S. Systematic literature review of access pathways to drugs for patients with rare diseases. Applied Health Economics and Health Policy; 2024. 10.1007/s40258-024-00939-4 39731657

[4] Degtiar I. A review of international coverage and pricing strategies for personalized medicine and orphan drugs. Health Policy. 2017;121(12):1240–8. 29033060 10.1016/j.healthpol.2017.09.005

[5] Rosenberg-Yunger ZRS, Daar AS, Thorsteinsdottir H, Martin DK. Priority setting for orphan drugs: an international comparison. Health Policy. 2011;100(1):25–34. 20961647 10.1016/j.healthpol.2010.09.008

[6] de Andrés-Nogales F, Cruz E, Calleja M, Delgado O, Gorgas MQ, Espín J, et al. A multi-stakeholder multicriteria decision analysis for the reimbursement of orphan drugs (FinMHU-MCDA study). Orphanet J Rare Dis. 2021;16(1):186. 33902672 10.1186/s13023-021-01809-1 PMC8073956

[7] Lopez-Bastida J, Ramos-Goni JM, Aranda-Reneo I, Taruscio D, Magrelli A, Kanavos P. Using a stated preference discrete choice experiment to assess societal value from the perspective of patients with rare diseases in Italy. Orphanet J Rare Dis. 2019;14(1):154. 31242905 10.1186/s13023-019-1126-1 PMC6595697

[8] Postma MJ, Noone D, Rozenbaum MH, Carter JA, Botteman MF, Fenwick E, Garrison LP. Assessing the value of orphan drugs using conventional cost-effectiveness analysis: is it fit for purpose? Orphanet J Rare Dis. 2022;17(1):157. 35382853 10.1186/s13023-022-02283-z PMC8981887

[9] United Nations. UN Resolution on Persons Living with a Rare Disease 2021 [Available from: https://www.rarediseasesinternational.org/un-resolution/

[10] Lancaster KJ. A new approach to consumer theory. J Polit Econ. 1966;74(2).

[11] Street DJ, Viney R. Design of discrete choice experiments. Oxford University Press; 2019.

[12] Hole AR, Kolstad JR. Mixed logit Estimation of willingness to pay distributions: a comparison of models in preference and WTP space using data from a health-related choice experiment. Empirical Economics. 2012;42(2):445–69.

[13] Weller BE, Bowen NK, Faubert SJ. Latent class analysis: A guide to best practice. J Black Psychol. 2020;46(4):287–311.

[14] StataCorp. In: Station C, editor. Stata statistical software: release 18. TX: StataCorp LLC; 2023.

[15] Yoo HI. lclogit2: an enhanced command to fit latent class conditional logit models. Stata J. 2020;20(2):405–25.

[16] Ride J, Goranitis I, Meng Y, LaBond C, Lancsar E. A reporting checklist for discrete choice experiments in health: the DIRECT checklist. PharmacoEconomics. 2024;42(10):1161–75. 39227559 10.1007/s40273-024-01431-6 PMC11405421

[17] Bourke SM, Plumpton CO, Hughes DA. Societal preferences for funding orphan drugs in the UK: A person trade off study. Value Health. 2017;20(9):A559–60. 10.1016/j.jval.2017.12.026 29753350

[18] Mentzakis E, Stefanowska P, Hurley J. A discrete choice experiment investigating preferences for funding drugs used to treat orphan diseases: an exploratory study. Health Econ Policy Law. 2011;6(3):405–33. 21205401 10.1017/S1744133110000344

[19] Bae EY, Lim MK, Lee B, Bae G. Who should be given priority for public funding? Health Policy. 2020;124(10):1108–14. 32651005 10.1016/j.healthpol.2020.06.010

[20] Linley WG, Hughes DA. Reimbursement decisions of the all Wales medicines strategy group: influence of policy and clinical and economic factors. PharmacoEconomics. 2012;30(9):779–94. 22676385 10.2165/11591530-000000000-00000

[21] Bae EY, Lim MK, Choi SE, Lee TJ. The public’s preference on the priorities in health care. Value Health. 2010;13(7):A534.

[22] Desser AS, Gyrd-Hansen D, Olsen JA, Grepperud S, Kristiansen IS. Societal views on orphan drugs: cross sectional survey of Norwegians aged 40 to 67. BMJ. 2010;341:c4715. 20861122 10.1136/bmj.c4715 PMC2944922

[23] Chim L, Salkeld G, Kelly PJ, Lipworth W, Hughes DA, Stockler MR. Community views on factors affecting medicines resource allocation: cross-sectional survey of 3080 adults in Australia. Aust Health Rev. 2019;43(3):254–60. 29669674 10.1071/AH16209

[24] Richardson J, Iezzi A, Chen G, Maxwell A. Communal sharing and the provision of Low-Volume High-Cost health services: results of a survey. PharmacoEconomics Open. 2017;1(1):13–23. 29442298 10.1007/s41669-016-0002-3 PMC5689032

[25] Ramalle-Gomara E, Ruiz E, Quinones C, Andres S, Iruzubieta J, Gil-de-Gomez J. General knowledge and opinion of future health care and non-health care professionals on rare diseases. J Eval Clin Pract. 2015;21(2):198–201. 25363689 10.1111/jep.12281

[26] Desser AS, Olsen JA, Grepperud S. Eliciting preferences for prioritizing treatment of rare diseases: the role of opportunity costs and framing effects. PharmacoEconomics. 2013;31(11):1051–61. 24114738 10.1007/s40273-013-0093-y

[27] National Institute for Health and Care Excellence. Citizens Council Report. Ultra orphan drugs. London, UK2004 [Available from: https://www.nice.org.uk/Media/Default/Get-involved/Citizens-Council/Reports/CCReport04UltraOrphanDrugs.pdf.

[28] Skedgel C, Henderson N, Towse A, Mott D, Green C. Considering severity in health technology assessment: can we do better? Value Health. 2022;25(8):1399–403. 35393254 10.1016/j.jval.2022.02.004

[29] Ubel PA. How stable are people’s preferences for giving priority to severely ill patients? Soc Sci Med. 1999;49(7):895–903. 10468394 10.1016/s0277-9536(99)00174-4

[30] Vásquez P, Hall L, Merlo G. Societal preferences in health technology assessments for rare diseases and orphan drugs: A systematic literature review of new analytic approaches. Value Health Reg Issues. 2024;44:101026. 39059264 10.1016/j.vhri.2024.101026

[31] Angelis A, Kanavos P. Value-Based assessment of new medical technologies: towards a robust methodological framework for the application of multiple criteria decision analysis in the context of health technology assessment. PharmacoEconomics. 2016;34(5):435–46. 26739955 10.1007/s40273-015-0370-z PMC4828475

