# Supplementary material for: Using a discrete choice experiments to explore societal preferences for valuing new drugs for rare diseases
Source: Orphanet J Rare Dis. 2025 Dec 29;20:631. doi: 10.1186/s13023-025-04141-0 (PMC12751238; doi:10.1186/s13023-025-04141-0)
Supplement: Supplementary file 1 — Supplementary Material 1 [file 13023_2025_4141_MOESM1_ESM.docx]

# Supplementary material

## DCE Survey: Drugs for rare diseases and assessment pathway until public reimbursement

### Survey Consent

**Access to drugs for rare diseases in Australia**

**About this survey**

Thank you for your interest in participating in this survey. Diseases that affect few people are known as ‘rare diseases’. Our research is intended to help us understand what people thinks matter when deciding if a drug for a rare disease represents good value. We are also interested in what decision makers should consider to ensure patients’ access to drugs for rare diseases in Australia.

We would also like to hear your views on the survey itself; this will help us to improve our survey for future use. The last part of the survey asks some general questions about you, such as your education. This information can’t be linked back to your name or anything else that could identify you but is important to our research because it will help us to investigate patterns and differences that could help us in understanding the choices people make.

**How long will this survey take?**

Overall, the survey should take about 15 to 20 minutes to complete.

**What sort of device do I need to complete this survey?**

The survey cannot be completed on a mobile phone due to some of its display functions. Please use another web enabled device such as a PC, laptop or tablet. Thank you.

**Do I have to take part in this research study?**

Participation in this study is voluntary. Even if you do commence the survey, you can opt out at any time by closing your browser before you reach the end.

**Are there any risks or inconvenience from participating in the survey?**

We don’t expect participation in this survey to cause you any distress or inconvenience. However, if you do feel distressed you can change your mind at any time and stop completing the survey without consequences.

**How do I consent to be part of the study?**

Submission of the online questionnaire is an indication of your consent. By continuing to answer the survey questions you consent to the research team collecting and using your answers for the research project.

**What will happen to my information?**

All of your answers will be treated confidentially and are anonymous. There is no identifying information that is collected as part of the study and the researchers will not have access to any information that can identify you. All data will be stored securely. Data collected will be used for the purpose of this research project and potentially in future research projects that aim to improve on the methods used to design questionnaires of this type.

**How will information collected be used?**

The results of this study will be shared with clinicians and researchers who are interested in how patients with rare diseases access treatment. They will also be published in peer reviewed journals and presented at research conferences.  In all instances, results from the survey will be published in a form that does not identify you.

**Who is conducting this research?**

This study is being led by Constanza Vargas. It is part of a broader doctoral research approved by the University of Technology Sydney Human Research Ethics Committee (UTS HREC ETH21-6090).

**What if I have concerns or a complaint?**

If you have any questions or concerns about this survey, please contact Constanza Vargas at [constanza.vargas@uts.edu.au](mailto:constanza.vargas@uts.edu.au) or the UTS Ethics Secretariat: Research.Ethics@uts.edu.au.

If you would like to talk to someone who is not connected with the research, you may contact the Research Ethics Officer on 02 9514 9772 or Research.ethics@uts.edu.au and quote this number ETH21-6090.

If you agree to be part of the research and to research data gathered from this survey to be published in a form that does not identify you, please click on the button **‘****I accept participating in this survey’**, if you do not accept, you can close the survey.

### Set the scene page

**How are drugs funded in Australia**

In Australia, drugs are primarily funded through the Pharmaceutical Benefits Scheme (PBS), a government program that subsidizes the cost of medicines. Through this subsidy, patients pay a minimal or no cost for medications, as the government covers the majority of the expense. Due to limited resources, not all drugs receive public funding; decisions are guided by Health Technology Assessment (HTA), a process that evaluates the value of new drugs to support equitable, efficient, and high-quality healthcare.

**The problem of rare diseases**

A rare disease affects more than 1 patient and less than 5 in 10,000 people. Most rare diseases, have a genetic origin affecting mainly children who often die before the age of 5. Rare diseases can be severe having a negative impact on the patient, family and carer’s quality of life. The natural course of life gets altered as well as the day-to-day life activities such as work, social engagement and participation, hobbies, and even basic self-care may become challenging, often necessitating the involvement of caregivers.

Small patient populations lead to limited knowledge of the condition and minimal research, resulting in few available treatments. When treatments do become available, they often enter the market at very high prices due to the small target population. These high costs make them unaffordable without public funding and pose a significant opportunity cost for health systems.

### Attributes page

**Factors considered by decision makers when funding new drugs**

There are multiple factors considered by decision makers when making recommendations of drugs. Among these factors:

- the confidence in the clinical evidence showing that the drug works.
- Whether alternative treatments are available for the same disease.
- the total cost to Government of the drug; and
- the size of the benefit to the patient, in terms of survival and quality of life.

### Survey 1 Vignette Page

**What influences treatment choices in rare diseases**

Imagine you are providing advice to the Government of Australia on which drug for a rare disease should be funded for public access at no or minimal cost to the patient.

Few patients are affected by this rare condition. The rare disease has a substantial impact on patients' quality of life, in particular patients require a full-time carer from early childhood, and the disease leads to death in 10 years without treatment.

Some of the terms are highlighted in green – hover your mouse or pointer over the highlighted words if you want to see more information about those terms.

You will now see 8 hypothetical questions. For each, you will be asked which drug you would choose. Each time, please read all of the information on the page before you make your choice.

Please remember there are no right or wrong answers.

### Choice scenario

**Actual Choice Scenario Survey 1- Example**

| **Remember this is the base case:**  The rare disease has a substantial impact on patients' quality of life, in particular patients require a full-time carer from early childhood, and the disease leads to death in 10 years without treatment. |
| --- |

Imagine you are providing advice to the Government of Australia on which drug for a rare disease should be funded for public access at no or minimal cost to the patient. Consider these two options:

| **Description** | **Drug 1** | **Drug 2** |
| --- | --- | --- |
| The degree of confidence in the clinical evidence is described as: | Not confident that it works as there is very limited data on efficacy and safety. | Confident that it works as there is strong evidence on efficacy and safety. |
| The extent to which alternative treatments for the rare disease are available. | Other treatments are available but are of limited effectiveness. | No other treatment options are available. |
| The total annual treatment cost to Government for all patients with the rare condition in Australia is: | $100 million | $200 million |
| Extent to which the drug increases survival for the average patient | Increases survival by 1 year | Increases survival by 5 years |
| The use of the new drug means that the patient: | Will have no problems doing their usual activities, and does not require a carer. | Will have slight problems doing their usual activities, and does not require a carer. |

Which option would you choose:

- Drug 1
- Drug 2

*TRANSITION PAGE*

*This page should be displayed after completing the first set of choices.*

Thank you.  You have just completed the first set of 8 questions.  **Please don’t log off**.

We will now ask you a few questions regarding the choice questions you just answered. Post DCE1 choice questions

1. Thinking about the 8 questions you just answered, please fill out the following rating table.

|  |  | | | | |
| --- | --- | --- | --- | --- | --- |
|  | Strongly disagree (1) | Disagree (2) | Neither agree nor disagree (3) | Agree (4) | Strongly Agree (5) |
| The language used in the questions was clear. |  |  |  |  |  |
| The questions were easy to understand. |  |  |  |  |  |
| The task was easy to complete. |  |  |  |  |  |

1. Thinking about the factors which varied in each of the 8 questions on what is important in a drug for a rare disease, which one was the **most** important when choosing between the options you saw? Respondents can choose only one here.

- The level of confidence of clinical evidence.
- The availability of other treatments for the same rare condition.
- The total annual cost to treat the condition in Australia.
- The extent to which the drug increases survival.
- The extend to which the drug increases the ability of patients in doing usual activities.

1. Thinking about the factors which varied in each of the 8 questions on what is important in a drug for a rare disease, which one was the **least** important when choosing between the options you saw? Respondents can choose only one here.

- The level of confidence of clinical evidence.
- The availability of other treatments for the same rare condition.
- The total annual cost to treat the condition in Australia.
- The extent to which the drug increases survival.
- The extend to which the drug increases the ability of patients in doing usual activities.

## Direct checklist

Table S1. The DIRECT checklist for discrete choice experiments.

| **Section**  **Item** | | **Page and paragraph** |
| --- | --- | --- |
| Purpose and rationale | |  |
| 1 | Describe the real-world context and decision-maker that the hypothetical choice context seeks to replicate or inform | Subheading: Survey development in Box 1. |
| 2 | Provide a rationale for using a DCE to answer the research question | Background: Lines 99-102. |
| Attributes and levels^a^ | |  |
| 3 | Describe how attributes and levels were derived (e.g. literature review, interviews, focus groups, expert input) | Subheading: Identification of relevant attributes (lines 127-157). |
| 4 | Provide the final list of attributes and levels | Table 1. |
| Experimental design | |  |
| 5 | Report the number of alternatives per choice set and whether they were labelled or unlabelled | Subheading DCE design (Line 174-175) |
| 6 | Describe response options (e.g. forced choice, opt-out, status quo) | Subheading DCE design (Line 169-170) |
| 7 | Describe the type of experimental design (e.g. orthogonal, D-efficient, Bayesian efficient, partial profile) | Subheading DCE design (paragraph 1 under this subheading) |
| 8 | Describe which effects are identified in the design (e.g. main effects, higher order interactions, functional form) | Subheading DCE design (Line 170-171) |
| 9 | Describe the number of choice sets, blocks and choice sets per block | Subheading DCE design (Lines 172-176) |
| 10 | Indicate how the experimental design was obtained (software, catalogue, other) | Subheading DCE design (Line 173-174) |
| Survey design | |  |
| 11 | Provide a sample choice set and the instructions and background information given to respondents (e.g. providing the survey as an appendix) | Figure 1 and complete survey provided in Supplementary material. |
| 12 | Report any randomisation (e.g. choice set order, attribute order, alternative order, framing effects) | Subheading DCE design (Line 174-176) |
| 13 | Describe what was checked in piloting (e.g. understanding, respondent burden, timing, wording) | Subheading Data collection (Line 191-195) |
| 14 | Report whether information from the pilot was used to update the experimental design (e.g. priors, functional form of attributes) or survey design | Subheading Data collection (Line 191-195) |
| Sample and data collection | |  |
| 15 | Report respondent inclusion/exclusion criteria | Subheading Data collection (Lines 184-188) |
| 16 | Describe how data were collected (e.g. mail, personal interview, web survey) | Subheading Data collection (Line 184) |
| 17 | Report the response rate or cooperation rate, if possible | This was not assessed. |
| 18 | Report the final sample size and how the sample size was determined | Subheading DCE design (195-196)  Results (Line 240) |
| 19 | Describe respondent characteristics and representativeness of target population, if known | Results (Lines 240-244)  Table S2 in the supplementary material. |
| Econometric analysis | |  |
| 20 | Indicate coding of data (e.g. effects, dummy, continuous) including definitions | Subheading Statistical analysis (Lines 221-226) |
| 21 | Report whether any respondents were removed and why (e.g. suspected fraudulent responses, rationality tests) | All respondents were included. |
| 22 | Provide the rationale for model choice (e.g. conditional logit, mixed logit, latent class) and assumptions (e.g. error variance) | Subheading Statistical analysis. |
| 23 | Report model specification | Subheading Statistical analysis (217-221) |
| Reporting of results | |  |
| 24 | Report the model performance, goodness of fit (if comparing models) | Not applicable. Goodness of fit of models are reported in the supplementary material. |
| 25 | Describe methods used for analysis of model results (e.g. calculation of marginal rate of substitution, attribute relative importance, welfare gain) | Subheading Statistical analysis (Lines 221-226) |
| 26 | Report measures of precision for the output(s) of interest (e.g. confidence intervals) and how these were derived | Table 2. |

^a^For reporting of formative qualitative analysis, see <https://www.equator-network.org/reporting-guidelines/using-qualitative-methods-for-attribute-development-for-discrete-choice-experiments-issues-and-recommendations/>

## Additional results

Table S2: Demographic characteristics of respondents

| **Sample characteristic** | **Sample N = 1099**  **n (%)** |
| --- | --- |
| **Sex n (%)** |  |
| Male, | 543 (49.41) |
| Female, | 556 (50.59) |
| **Age, n (%)** |  |
| 18-24 | 121 (11.01) |
| 25-34 | 191 (17.38) |
| 35-44 | 194 (17.65) |
| 45-54 | 183 (16.65) |
| 55-64 | 168 (15.29) |
| > 65 years | 242 (22.02) |
| **State** |  |
| NSW | 350 (31.9) |
| VIC | 283 (25.8) |
| QLD | 218 (19.8) |
| SA | 80 (7.3) |
| WA | 115 (10.5) |
| ACT | 25 (2.3) |
| TAS | 19 (1.7) |
| NT | 9 (0.8) |
| **Highest level of education** |  |
| Finished school before Year 10 | 23 (2.09) |
| Year 10 or equivalent | 74 (6.73) |
| Year 12 or equivalent | 215 (19.56) |
| Trade/apprenticeship | 240 (21.84) |
| Undergraduate university degree | 345 (31.39) |
| Postgraduate university degree | 197 (17.93) |
| Prefer not to say | 5 (0.45) |
| **Income per year** |  |
| Negative or zero Income | 11 (1) |
| $1 - $9,999 | 15 (1.36) |
| $10,000 - $19,999 | 24 (2.18) |
| $20,000 - $29,999 | 63 (5.73) |
| $30,000 - $39,999 | 70 (6.37) |
| $40,000 - $49,999 | 67 (6.10) |
| $50,000 - $59,999 | 82 (7.46) |
| $60,000 - $79,999 | 104 (9.46) |
| $80,000 - $99,999 | 117 (10.65) |
| $100,000 - $124,999 | 125 (11.37) |
| $125,000 - $149,999 | 103 (9.37) |
| $150,000 - $199,999 | 138 (12.56) |
| ≥$200,000 | 103 (9.37) |
| Prefer not to say | 67 (6.1) |
| Don't know | 10 (0.91) |
| **Health status** |  |
| Excellent | 116 (10.56) |
| Very good | 374 (34.03) |
| Good | 425 (38.67) |
| Fair | 157 (14.29) |
| Poor | 27 (2.46) |
| **Employment status** |  |
| Full time | 502 (45.68) |
| Part time | 184 (16.74) |
| Casual | 52 (4.73) |
| Home duties | 46 (4.19) |
| Studying | 24 (2.18) |
| Unemployed | 53 (4.82) |
| Parental leave | 3 (0.27) |
| Retired | 229 (20.84) |
| Prefer not to say | 6 (0.55) |
| **Relation status** |  |
| Married | 522 (47.5) |
| Long-term relationship | 140 (12.74) |
| Separated | 28 (2.55) |
| Divorced | 77 (7.01) |
| Widowed | 29 (2.64) |
| Single | 296 (26.93) |
| Prefer not to say | 7 (0.64) |
| **Do you have children?** |  |
| No | 437 (39.76) |
| Yes | 653 (59.42) |
| Prefer not to say | 9 (0.82) |
| **Have you or a family member been prescribed a high-cost drug** |  |
| No | 952 (86.62) |
| Yes, I needed a high-cost treatment | 78 (7.1) |
| Yes, a family member required a high-cost treatment. | 54 (4.91) |
| Prefer not to say | 15 (1.36) |
| **Carer** |  |
| Yes | 97 (8.83) |
| No | 982 (89.35) |
| Prefer not to say | 20 (1.82) |
| **Experience with rare diseases** |  |
| No | 955 (86.9) |
| Yes – I have a relative/friend with a rare disease | 124 (11.28) |
| Yes – I have a rare disease | 19 (1.73) |
| Both my child and I have a rare disease | 1 (0.09) |

|  |
| --- |

Figure S1 Results of the conditional logit model

Figure S2 Results of the conditional logit model with respondents taking between 0.96 and 7.69 minutes.

Table S2 Latent class model to identify number of classes

| Model | ll(null) | ll(model) | df | CAIC | BIC | AIC |
| --- | --- | --- | --- | --- | --- | --- |
|  |  |  |  |  |  |  |
| 2 Classes | . | -4738.8 | 32 | 9822.393 | 9790.393 | 9541.601 |
| **3 Classes** | **.** | **-4593.22** | **53** | **9757.498** | **9704.498** | **9292.436** |
| 4 Classes | . | -4492.97 | 74 | 9783.27 | 9709.27 | 9133.939 |
| 5 Classes | . | -4440.39 | 95 | 9904.382 | 9809.382 | 9070.782 |
| 6 Classes | . | -4421.48 | 116 | 10092.83 | 9976.826 | 9074.955 |
| 7 Classes | . | -4388.73 | 137 | 10253.61 | 10116.61 | 9051.468 |
| 8 Classes |  | Convergence not achieved | | |  |  |
| 9 Classes |  | Convergence not achieved | | |  |  |
| 10 Classes | | Convergence not achieved | | |  |  |

Table S3 Latent class analysis results

| prefsDCE1 | Coefficient | Std. err. | z | P>z | [95% conf. | interval] |
| --- | --- | --- | --- | --- | --- | --- |
| Class1 |  |  |  |  |  |  |
| Confident | 0.229134 | 0.075968 | 3.02 | 0.003 | 0.08024 | 0.378029 |
| Limitedeffectiveness | 0.072283 | 0.091154 | 0.79 | 0.428 | -0.10638 | 0.250942 |
| NoTxavailable | -0.11009 | 0.100205 | -1.1 | 0.272 | -0.30649 | 0.086309 |
| Survive1year | 0.150075 | 0.120182 | 1.25 | 0.212 | -0.08548 | 0.385627 |
| Survive5years | 0.275571 | 0.130274 | 2.12 | 0.034 | 0.020239 | 0.530904 |
| Moderateproblems | 0.154407 | 0.126542 | 1.22 | 0.222 | -0.09361 | 0.402425 |
| Slightproblems | 0.298207 | 0.151067 | 1.97 | 0.048 | 0.002121 | 0.594293 |
| Noproblems | 0.369116 | 0.149451 | 2.47 | 0.014 | 0.076197 | 0.662035 |
| Cost50M | 0.186711 | 0.117 | 1.6 | 0.111 | -0.0426 | 0.416026 |
| Cost100M | 0.409957 | 0.139325 | 2.94 | 0.003 | 0.136884 | 0.68303 |
| Cost200M | 0.326599 | 0.145923 | 2.24 | 0.025 | 0.040595 | 0.612602 |
|  |  |  |  |  |  |  |
| Class2 |  |  |  |  |  |  |
| Confident | 3.41388 | 0.342803 | 9.96 | 0 | 2.741997 | 4.085762 |
| Limitedeffectiveness | 0.569371 | 0.226823 | 2.51 | 0.012 | 0.124807 | 1.013935 |
| NoTxavailable | 0.1798 | 0.254336 | 0.71 | 0.48 | -0.31869 | 0.678289 |
| Survive1year | 0.730289 | 0.288544 | 2.53 | 0.011 | 0.164753 | 1.295824 |
| Survive5years | 1.291235 | 0.256459 | 5.03 | 0 | 0.788584 | 1.793885 |
| Moderateproblems | 0.721288 | 0.260245 | 2.77 | 0.006 | 0.211217 | 1.231358 |
| Slightproblems | 1.193253 | 0.314026 | 3.8 | 0 | 0.577774 | 1.808732 |
| Noproblems | 1.784197 | 0.321826 | 5.54 | 0 | 1.15343 | 2.414964 |
| Cost50M | -0.50464 | 0.248095 | -2.03 | 0.042 | -0.9909 | -0.01839 |
| Cost100M | -0.79712 | 0.369601 | -2.16 | 0.031 | -1.52153 | -0.07272 |
| Cost200M | -0.9122 | 0.351363 | -2.6 | 0.009 | -1.60086 | -0.22354 |
|  |  |  |  |  |  |  |
| Class3 |  |  |  |  |  |  |
| Confident | 0.885823 | 0.069454 | 12.75 | 0 | 0.749697 | 1.021949 |
| Limitedeffectiveness | -0.12364 | 0.065657 | -1.88 | 0.06 | -0.25232 | 0.00505 |
| NoTxavailable | -0.01522 | 0.073916 | -0.21 | 0.837 | -0.16009 | 0.129657 |
| Survive1year | 0.91937 | 0.086585 | 10.62 | 0 | 0.749667 | 1.089074 |
| Survive5years | 2.227874 | 0.125845 | 17.7 | 0 | 1.981222 | 2.474526 |
| Moderateproblems | 0.795933 | 0.089142 | 8.93 | 0 | 0.621219 | 0.970648 |
| Slightproblems | 1.165194 | 0.102677 | 11.35 | 0 | 0.963951 | 1.366436 |
| Noproblems | 1.41978 | 0.106741 | 13.3 | 0 | 1.210572 | 1.628988 |
| Cost50M | -0.77223 | 0.090273 | -8.55 | 0 | -0.94916 | -0.5953 |
| Cost100M | -1.45802 | 0.111193 | -13.11 | 0 | -1.67596 | -1.24009 |
| Cost200M | -2.01313 | 0.134694 | -14.95 | 0 | -2.27713 | -1.74914 |
| Memberships |  |  |  |  |  |  |
| Share1 |  |  |  |  |  |  |
| gender | 0.202317 | 0.218635 | 0.93 | 0.355 | -0.2262 | 0.630834 |
| health_status | 0.354841 | 0.318649 | 1.11 | 0.265 | -0.2697 | 0.979381 |
| health_literacy | -1.04542 | 0.469507 | -2.23 | 0.026 | -1.96563 | -0.1252 |
| decision_maker | -0.00098 | 0.247262 | 0 | 0.997 | -0.4856 | 0.483647 |
| income | -0.34684 | 0.22894 | -1.52 | 0.13 | -0.79556 | 0.10187 |
| education | -0.57621 | 0.235383 | -2.45 | 0.014 | -1.03756 | -0.11487 |
| rare | 0.076904 | 0.320693 | 0.24 | 0.81 | -0.55164 | 0.705451 |
| children | -0.44806 | 0.218786 | -2.05 | 0.041 | -0.87688 | -0.01925 |
| carer | 0.9193 | 0.344123 | 2.67 | 0.008 | 0.244832 | 1.593768 |
| _cons | 0.229593 | 0.679477 | 0.34 | 0.735 | -1.10216 | 1.561345 |
|  |  |  |  |  |  |  |
| Share2 |  |  |  |  |  |  |
| gender | 0.121612 | 0.204561 | 0.59 | 0.552 | -0.27932 | 0.522544 |
| health_status | -0.12218 | 0.273279 | -0.45 | 0.655 | -0.6578 | 0.413438 |
| health_literacy | 0.810275 | 0.843979 | 0.96 | 0.337 | -0.84389 | 2.464444 |
| decision_maker | 0.070327 | 0.229763 | 0.31 | 0.76 | -0.38 | 0.520655 |
| income | 0.644773 | 0.250076 | 2.58 | 0.01 | 0.154634 | 1.134913 |
| education | 0.01173 | 0.240495 | 0.05 | 0.961 | -0.45963 | 0.483091 |
| rare | 0.023052 | 0.302839 | 0.08 | 0.939 | -0.5705 | 0.616605 |
| children | -0.09256 | 0.20367 | -0.45 | 0.649 | -0.49175 | 0.306623 |
| carer | -0.59423 | 0.570001 | -1.04 | 0.297 | -1.71142 | 0.522947 |
| _cons | -2.24632 | 0.968948 | -2.32 | 0.02 | -4.14542 | -0.34722 |
